# Implicit Theories and Engagement in Math Among Chinese Adolescent Students: A Moderated Mediation Model of Intrinsic Value and Academic Self-Efficacy

**DOI:** 10.3389/fpsyg.2020.01325

**Published:** 2020-06-26

**Authors:** Shuyang Jiang, Ru-De Liu, Yi Ding, Xinchen Fu, Yan Sun, Ronghuan Jiang, Wei Hong

**Affiliations:** ^1^Beijing Key Laboratory of Applied Experimental Psychology, National Demonstration Center for Experimental Psychology Education (Beijing Normal University), Faculty of Psychology, Beijing Normal University, Beijing, China; ^2^Graduate School of Education, Fordham University, New York, NY, United States

**Keywords:** implicit theories, math engagement, intrinsic value, self-efficacy, adolescents

## Abstract

Recent studies have established associations between students’ implicit theories and their academic engagement. However, there is still limited understanding of the potential mechanisms of this relation, and whether it works for students in the context of mathematics as well as in other subjects. The current study aimed to fill this gap by conducting a two-wave survey examining a moderated mediation model concerning the psychological mechanisms that account for the association between students’ implicit theories and mathematics engagement. Applying the theoretical framework of implicit theory, we hypothesized that intrinsic value would be a possible mediating variable between students’ implicit theories and academic engagement, and that students’ academic self-efficacy would moderate the link between implicit theory and intrinsic value. A sample of 710 Chinese adolescent students self-reported their implicit theory, intrinsic value, and academic self-efficacy at Time 1, and engagement in math at Time 2, 12 months apart. After controlling for age and gender, the results revealed positive associations between students’ implicit theories and their engagement in math, and intrinsic value partially mediated the relation between implicit theories and engagement in math. Moreover, students’ academic self-efficacy moderated the link between implicit theory and intrinsic value. These findings contribute to the understanding of the impact of implicit theory on students’ mathematics engagement. Limitations and implications for instructional practices are discussed.

## Introduction

Academic engagement has been recognized as a key indicator in school achievement and assessment with the focus on the extent to which students are willing to invest their time and effort in academic domains, such as math and science (Pintrich and Schunk, 1996; Fredricks et al., 2004). It has been well-documented that academic engagement not only predicts school achievement and various adaptation outcomes (Fredricks et al., 2017), but also is critical for developing the fundamental skills and qualities necessary for pursuing college majors and future careers (Maltese and Tai, 2010).

In view of the significance of academic engagement, many researchers have examined the factors that might predict academic engagement to keep students motivated to learn, especially in math. Math is one of the key STEM fields that provide impetus for societal and economic growth (Preacher et al., 2006). Greater participation of qualified higher education graduates is the basis for the development of math-related technologies (Wang, 2013). However, mathematics is also considered as one of the most important and difficult academic subjects (Dündar et al., 2014), and recent researchers have indicated a low percentage of students pursuing mathematics-based courses, with mathematics engagement declining as students mature (Martin et al., 2015; Wigfield et al., 2015). These studies emphasize the urgent need to facilitate students’ engagement in math (Brown et al., 2008; Martin et al., 2015). Based on these findings, the current study will examine potential predictors and underlying mechanisms of mathematics engagement to further promote students’ long-term interest and participation in mathematics.

### Implicit Theory and Engagement in Math

Implicit theory is a belief that people hold regarding whether abilities are fixed or changeable (Dweck and Leggett, 1988). Previous studies have considered implicit theory as a unidimensional construct, with the incremental and entity theories resting on opposite extremes of a continuum (see Molden and Dweck, 2006 for a review). Those who endorse the incremental theory believe that ability can be improved through education and practice; in contrast, those who endorse the entity theory believe that ability cannot or can hardly be affected by efforts (Blackwell et al., 2007). Dweck’s theoretical framework has proposed that differences in implicit theory may lead to different goal orientation, achievement motivation, and responses to difficulties and setbacks, which in turn can affect academic outcomes (Mangels et al., 2006; Molden and Dweck, 2006; Blackwell et al., 2007; Dinger et al., 2013).

Researchers have consistently provided evidence indicating that implicit theory plays a critical role in students’ academic engagement. Higher endorsement of the incremental theory significantly predicted learning goals (Dinger and Dickhäuser, 2013), more positive studying strategies such as using multiple methods to solve academic tasks (Jones et al., 2012; Bodill and Roberts, 2013), better self-regulation (Mouratidis et al., 2016), and fewer procrastination behaviors (Howell and Buro, 2009). In a meta-analysis, Burnette et al. (2013) found that different implicit theories led to distinct orientations in terms of goal setting, operating, and monitoring processes. Similar results have been found in the learning of mathematics. Priess-Groben and Hyde (2017) found that after controlling for prior mathematics achievement, implicit theories could predict course-taking intentions and utility value. Rattan et al. (2012) also suggested that instructors who held a fixed theory of math intelligence tended to ascribe students’ low performance to low math abilities and were more likely to adopt fewer effort-oriented strategies. Taken together, these findings suggest that students who endorse more incremental beliefs are more likely to spend more time and effort in academic tasks, experience more positive emotions, and use more effective learning strategies, hence enhancing their engagement in math.

While researchers have shown that implicit theory may be associated with students’ engagement in math, most of these studies focused on students’ implicit theories from a general perspective, which limited the understanding of the relationship between implicit theory and engagement in specific domains. In fact, students’ implicit theories can vary across academic domains (Dweck, 2000), and compared to general beliefs, domain-specific beliefs were found to be a stronger predictor of students’ learning behaviors and achievement (Shively and Ryan, 2013; Gunderson et al., 2017; Priess-Groben and Hyde, 2017; Costa and Faria, 2018). More importantly, in China, students’ academic performance (e.g., test scores) is the primary standard not only for regular assessment but also for college admission. With such a strong focus on academic performance that is reflected through grades, implicit theory may play a unique role in mathematics engagement. Thus, the present study aims to elucidate the underlying mechanism of the relationship between implicit theory and engagement in math.

### Intrinsic Value as a Mediator

Previous research has suggested that intrinsic value may be a mediator in the link between implicit theory and engagement. Intrinsic value concerns students’ subjective beliefs about the importance and enjoyment of engaging in academic tasks (Eccles, 2009). Drawing upon the theoretical framework of implicit theories, we hypothesized two reasons for the positive relationship between incremental theory and intrinsic value. First, we postulated that higher endorsement of incremental theory can enhance students’ expectation to develop their abilities, which further promotes students’ evaluation of the importance of academic tasks. For example, incremental theory helps students to adopt a learning-goal orientation, in which students are more likely to consider academic tasks as valuable opportunities to learn new knowledge and master skills (Heslin et al., 2005). In contrast, a higher endorsement of entity theory can lead to a performance-goal orientation in which the students are more threatened by academic challenges, since they might consider that even the smallest setback can reflect their low abilities (Blackwell et al., 2007; Jones et al., 2012). Second, implicit theory can have an impact on students’ interests and emotional experiences. When encountering academic failure, students who endorse higher levels of incremental theory are more likely to attribute their low performance to lack of effort, maintain expectations toward future achievement, and experience fewer negative emotions (Dweck, 2000, 2002; Blackwell et al., 2007). In contrast, students with low levels of incremental theory tend to attribute academic failures to their low abilities, which are essentially unchangeable. They may lose control of their own academic performance and feel unintelligent, frustrated, and hopeless, which can lead to helpless-oriented reactions (Diener and Dweck, 1980), thus resulting in low intrinsic motivation.

Students who intrinsically value academic tasks are motivated to put forth more effort and show greater persistence and enjoyment in academic activities (Eccles, 1983; Eccles and Wigfield, 2002; Spinath et al., 2006; Wigfield and Cambria, 2010; Federici and Skaalvik, 2014). Wang et al. (2017) found that Chinese middle-school students with higher intrinsic value tended to be more engaged in mathematics. In summary, drawing on perspectives and empirical evidence based on implicit theory, we hypothesized that intrinsic value would mediate the association between implicit theory and academic engagement among students.

### Academic Self-Efficacy as a Moderator

Given that researchers have demonstrated a positive link between incremental theory and learning processes (e.g., motivation, effort), some studies have focused on improving students’ intrinsic motivation and engagement by means of interventions based on incremental theory, and such studies have indicated positive results (Levy et al., 1998; Aronson et al., 2002; Rattan et al., 2012). However, it remains unclear whether the benefits of incremental theory apply equally to students with different academic status. We anticipated that the relations between implicit theory and intrinsic value may depend on the students’ academic self-efficacy.

Academic self-efficacy refers to personal beliefs regarding an individual’s capabilities to succeed or accomplish goals in specific domains (Bandura, 1982). We hypothesized that students’ academic self-efficacy would moderate the effect of incremental beliefs on intrinsic value. That is, the lower the individuals’ evaluation of their academic self-efficacy, the greater the impact of incremental theory on intrinsic values. Convincing evidence suggests that students with low academic self-efficacy may benefit more from incremental beliefs. One of the key points of incremental beliefs is that they can reduce the negative influence of academic failures (Dweck, 2002). Students with low academic self-efficacy are exposed to more failure conditions than students with high academic self-efficacy, which provides an opportunity for incremental theory to fully play its role. To be specific, students with incremental beliefs tend to attribute academic failures to lack of effort; thus, they believe their present achievements do not reflect their actual abilities, and differences can be attained through hard work. Similar results have been demonstrated in previous studies. For example, a study by Froehlich et al. (2016) showed that fixed beliefs enhanced vulnerability to negatively stereotyped conditions and exerted a performance boost to favorably stereotyped conditions. It is thus reasonable to expect that students’ academic self-efficacy has an impact on the function of implicit theory in intrinsic value.

### The Current Study

**FIGURE 1:**
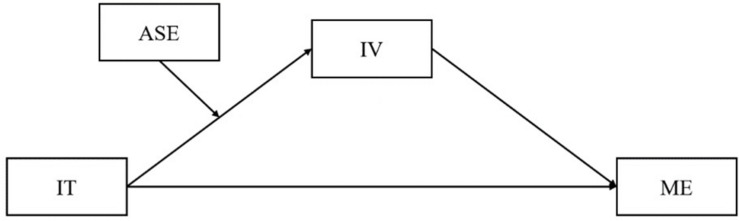
The hypothesized model. IT, implicit theory; IV, intrinsic value; ME, math engagement; ASE, academic self-efficacy.

The present study filled the gap in previous research to examine psychological mechanisms through which implicit theory is associated with math engagement among Chinese adolescent students. Our first purpose was to examine whether intrinsic value plays a mediating role in the relation between implicit theory and math engagement. In addition, we tested whether students’ academic self-efficacy moderated the direct association between implicit theory and intrinsic value. This study will promote our understanding of factors and pathways to impact students’ math engagement and provide evidence for effective educational practice. The model that was tested is presented in Figure 1.

## Materials and Methods

### Participants and Procedure

The current study was approved by the Research Ethics Committee of a major research university in China and the principals of the participating schools. Since all the participants are adolescents, individual informed consent to participate in the study was collected from both children and their parents, along with written consent describing the purpose and voluntary nature of the study.

Previous studies have indicated that one-wave design of mediation is potentially biased due to common method variance (Podsakoff et al., 2003). Therefore, we planned to collect data at two time points to reduce possible common method biases and improve methodological rigor in testing our model (Wright et al., 2005; see Haller et al., 2018; Zhuang et al., 2018 for a similar approach). At the first wave assessment (T1), 370 students from Grade 8 and 369 students from Grade 11 completed the measures of implicit theory, academic self-efficacy, and intrinsic value. Twelve months later, the measure of engagement in math was distributed to those participants (T2). With the cooperation of the school, 99.8% of the first-wave participants were retained; the attrition occurred mainly because of student absences on the day of assessment. As a result, the final sample was composed of 364 eighth-grade (172 males, mean age 13.0 years) and 346 eleventh-grade (166 males, mean age 16.4 years) students.

### Measures

#### Implicit Theory

We adopted an implicit theory of ability scale to assess students’ implicit belief in math learning. The scale is a four-item instrument (Dweck et al., 1995) that we modified to fit the math context. A sample item was, “Your math ability is something about you that you can’t change” (reverse-coded). Participants responded on a 5-point Likert-type scale ranging from 1 (*strongly disagree*) to 5 (*strongly agree*). This scale demonstrated adequate internal reliability in the present study (α = 0.82). Note that implicit theory has been considered as a unidimensional structure, with a higher score indicating a greater endorsement of the incremental theory, and a lower endorsement of the entity theory (see Davis et al., 2011; King et al., 2012; Tarbetsky et al., 2016 for a similar approach).

#### Academic Self-Efficacy

Academic self-efficacy was measured using the academic self-efficacy subscale from the Motivational and Self-regulated Learning Questionnaire (MSLQ). The original questionnaire was compiled by Pintrich and de Groot (1990), and we adopted the Chinese version. The subscale included nine items; a sample item was, “I expect to do very well in math class.” Participants responded on a 5-point Likert-type scale ranging from 1 = *not true at all of me* to 5 = *very true of me*. The scale had good internal reliability in the present study (α = 0.90).

#### Intrinsic Value

The nine-item intrinsic value subscale from the MSLQ (Pintrich and de Groot, 1990) was used to measure the students’ intrinsic value in math learning. We revised this scale to fit the math context (e.g., “I think what we are learning in math class is interesting.”). Students were asked to respond on a 5-point Likert-type scale ranging from 1 = *not true at all of me* to 5 = *very true of me*. In this study, the scale had satisfactory internal consistency (α = 0.85).

#### Engagement in Math

We assessed students’ engagement in math by means of the Math and Science Engagement Scales, originally developed by Wang et al. (2016). In the current study, the Chinese version revised by Liu et al. (2018) was used to ensure applicability to Chinese students. The scale consisted of three subscales: cognitive engagement (e.g., “When I study math, I try to connect what I am currently learning with the knowledge I have learned in the past.”), behavioral engagement (e.g., “I finish my math homework on time.”), and emotional engagement (e.g., “I enjoy learning new knowledge about math.”). Prior research confirmed both the reliability and the validity of this scale (Liu et al., 2018). The Cronbach’s alpha reliability coefficient of the overall math engagement scale was 0.89.

### Data Analytical Strategies

Before testing the hypotheses, missing values were filled by the Expectation Maximization (EM) method using the SPSS 23.0 software. We conducted the Harman’s one-factor test to examine common method variance (Podsakoff and Organ, 1986). Descriptive analysis, Cronbach’s alpha reliability coefficients, and the Pearson correlations coefficients between main variables were computed.

SPSS macro PROCESS was utilized to test the proposed hypotheses. The SPSS PROCESS macro was developed by Hayes (2013) and was widely used for testing complex models that include both mediating and moderating effects (e.g., Górnik-Durose and Boroń, 2018). Before conducting analysis, Process Macro^1^ was installed onto Regression of the SPSS software. Then, following the templates of preprogrammed models contained in Process, a three-step procedure was conducted to examine the moderated mediation model of academic self-efficacy and intrinsic value in the relation between implicit theories and engagement in math. First, to test the mediation model, we used the bootstrapping method with PROCESS macro (model 4) to calculate the 95% confidence intervals with 5,000 resamples. After controlling for age and gender, we developed a model to assess the mediating effect of intrinsic value in the relation between implicit theories and math engagement. Indirect path coefficients, of which the 95% confidence interval does not include zero, are considered statistically significant. Second, we used PROCESS macro (model 7) to test the moderated mediation model. Finally, to further reveal the nature of the interaction effects, a simple slopes method was employed to plot the conditional indirect effects (Hayes and Matthes, 2009; Jose, 2013).

## Results

### Common Method Variance Analysis

First, before testing the hypotheses, we assessed the common method variance (CMV) by conducting the Harman’s one-factor test. According to Podsakoff and Organ (1986), if the one general factor accounts for more than 40% of the total variance, it indicates the presence of a common method variance. In this study, the EFA results showed 10 factors with eigenvalues exceeding 1, and the first factor explained 26.30% of the total variance, which indicated that common method variance was not a serious concern in the present study.

### Descriptive and Correlation Analyses

Descriptive analysis and Pearson correlations analysis for the main variables are presented in Table 1. We found that age was negatively associated with implicit theory, academic self-efficacy, intrinsic value, and math engagement. In addition, gender was found to have a significant negative relation with implicit theory, academic self-efficacy, and intrinsic value. Except for age and gender, correlations between all the other main variables were significant and positive.

**TABLE 1:** Means, standard deviations, and correlations among the main variables.

	*M* ± *SD*	Range	1	2	3	4	5	6
1. T1 Age	14.60 ± 1.73	11, 18	–					
2. Gender	–	–	–	–				
3. T1 Implicit theory	2.48 ± 0.98	0.00, 4.00	−0.10*	−0.16***	–			
4. T1 Academic self-efficacy	3.21 ± 0.90	1.00, 5.00	−0.15***	−0.29***	0.38***	–		
5. T1 Intrinsic value	3.54 ± 0.80	1.00, 5.00	−0.20***	−0.11**	0.31***	0.39***	–	
6. T2 Math engagement	3.06 ± 0.63	1.03, 4.56	−0.33***	–0.07	0.28***	0.42***	0.47***	–

Gender was coded as 1 = males; 2 = females; T1 = Time 1; T2 = Time 2, ***p < 0.001; **p < 0.01; *p < 0.05.

### Mediation Analyses

**FIGURE 2:**
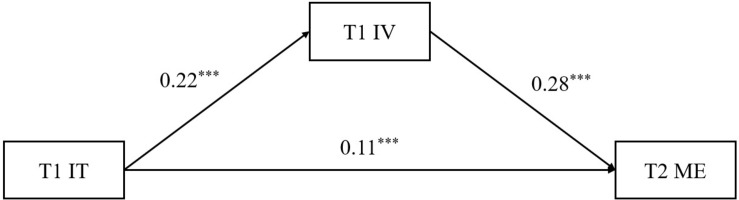
The mediating effects model after controlling for age and gender. IT, implicit theory; IV, intrinsic value; ME, math engagement; T1, Time 1; T2, Time 2, ***p < 0.001.

To test the mediation model, we placed T1 intrinsic value in the relation between T1 implicit theory and T2 math engagement. The PROCESS macro was used to examine the model shown in Figure 2.

The results revealed that there was a positive significant effect of T1 implicit theory on T1 intrinsic value (path a; β = 0.22, *p* < 0.001, 95% CI [0.15, 0.29]) and T1 intrinsic value on T2 math engagement (path b; β = 0.28, *p* < 0.001, 95% CI [0.22, 0.34]). The indirect effect was also significant (a × b; β = 0.06, 95% CI [0.04, 0.09]). Moreover, after we inserted T1 intrinsic value into the relation between T1 implicit theory and T2 math engagement, the direct effect remained significant (path c’; β = 0.11, 95% CI [0.06, 0.16]), which indicated a partial mediation.

### Moderated Mediation Analyses

**FIGURE 3:**
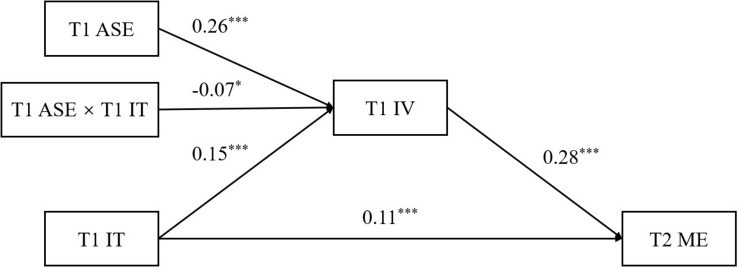
The moderated mediation model prediction after controlling for age and gender. IT, implicit theory; IV, intrinsic value; ME, math engagement; T1, Time 1; T2, Time 2, ***p < 0.001; *p < 0.05.

We conducted moderated mediation analysis to examine the model shown in Figure 3. The results indicated that the direct effect on T2 math engagement appeared for T1 implicit theory (β = 0.11, *p* < 0.001, 95% CI [0.05, 0.15]) and for T1 intrinsic value (β = 0.28, *p* < 0.001, 95% CI [0.22, 0.34]). In addition, the main effect of T1 implicit theory on T1 intrinsic value was significant (β = 0.15, *p* < 0.001, 95% CI [0.08, 0.21]), as well as the main effect of T1 academic self-efficacy (β = 0.26, *p* < 0.001, 95% CI [0.18, 0.34]). The interaction effect of T1 implicit theory and T1 academic self-efficacy on T1 intrinsic value was also significant (β = −0.07, *p* < 0.05, 95% CI [−0.13, −0.01]).

The indirect effect of T1 intrinsic value in the relation between T1 implicit theory and T2 math engagement indicated that T1 implicit theory was significantly positively related to T2 math engagement through T1 intrinsic value at low T1 academic self-efficacy (β = 0.05, 95% CI [0.03, 0.09]), but was non-significantly related with high T1 academic self-efficacy (β = 0.02, 95% CI [−0.00, 0.05]).

**FIGURE 4:**
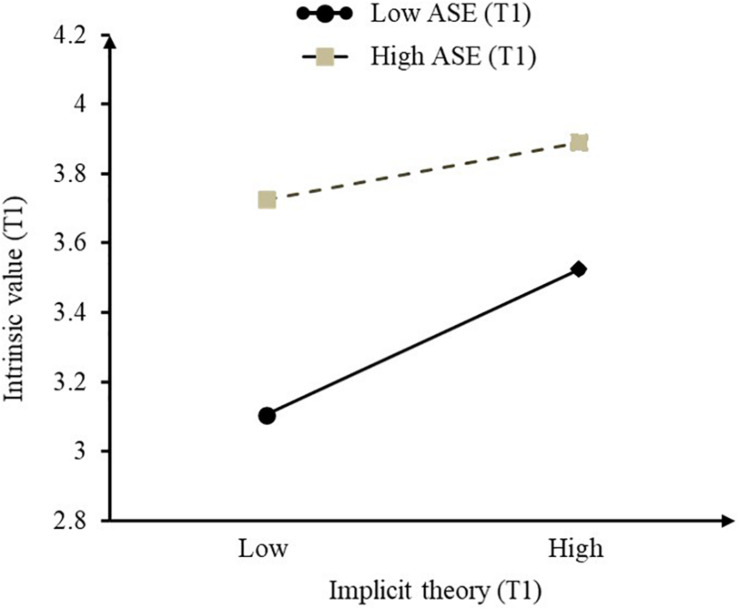
The interaction of T1 implicit theory and T1 academic self-efficacy on T1 intrinsic value. T1, Time 1.

To further reveal the pattern of the interaction, we plotted the conditional effects of T1 implicit theory on T1 intrinsic value, at ± 1 SD levels of T1 academic self-efficacy, respectively (see Figure 4). The findings suggested that the conditional effects of T1 implicit theory on students’ T1 intrinsic value were significantly larger for students with low T1 academic self-efficacy. With a low level of academic self-efficacy, implicit theory could predict students’ intrinsic value (β = 0.22, *p* < 0.001). However, with a high level of academic self-efficacy, the prediction of implicit theory failed (β = 0.083, *p* = 0.055 > 0.05).

To conclude, the results confirmed the second hypothesis. Students’ T1 academic self-efficacy moderated the mediating effect of intrinsic value between T1 implicit theory and T2 math engagement.

## Discussion

Although previous investigations have explored the influence of implicit theory on learning processes (e.g., goal orientation, learning strategies), the current study contributes to the literature by examining the underlying mechanisms between implicit theory and students’ math engagement via a two-wave design, using a sample of Chinese adolescents. In the present study, we tested a moderated mediation model in which intrinsic value mediated the association between implicit theory and students’ engagement in math, and academic self-efficacy moderated the direct association between implicit theory and intrinsic value.

### Direct Associations of Implicit Theory With Mathematics Engagement

As the results showed, incremental beliefs of math ability have a significant positive effect on students’ self-reported mathematics engagement, which is consistent with our hypothesis and concurs with previous research (Martin et al., 2013; Mouratidis et al., 2016). Incremental beliefs can help students focus on solving problems and achieving mastery skills in mathematics; thus, individuals will be more confident to face challenging situations (Cury et al., 2008; Doron et al., 2009). Incremental beliefs can promote various forms of engagement, such as more problem-focused coping strategies (e.g., seeking social support for emotional and instrumental reasons; Doron et al., 2009), more efforts toward study (Jones et al., 2012; Mouratidis et al., 2016), more positive academic emotions (King et al., 2012), less procrastination (Howell and Buro, 2009), and higher levels of class participation (Martin et al., 2013).

### Mediating Effect of Intrinsic Value

According to the results of the mediation analysis, intrinsic value seems to play a mediating role in the link between implicit theory and mathematics engagement. This is consistent with our hypothesis and supports the expectancy-value theory perspective (Eccles and Wigfield, 2002). In fact, incremental beliefs may increase students’ expectancy for future success in mathematics (Burnette et al., 2013), which further enhances their perceived importance of learning. To be specific, when a student who believes that his or her math ability can be promoted through learning and practice is confronted with challenging mathematics tasks, he or she may have higher expectancy and lower ego threat and find math more interesting and enjoyable to learn. High intrinsic value acts as the fuel of self-driven learning behaviors, which facilitate students to persist longer (Wigfield and Eccles, 2002), invest more effort (Federici and Skaalvik, 2014), and achieve higher levels of mathematics engagement (Zhen et al., 2018).

### Moderating Effect of Academic Self-Efficacy

With regard to academic self-efficacy, the present findings showed that the positive association between implicit theories and intrinsic value was stronger for students with low academic self-efficacy in comparison to students with high academic self-efficacy. Specifically, higher incremental beliefs were associated with higher levels of intrinsic value only among students with low academic self-efficacy; however, this effect was not significant among students with high academic self-efficacy. These findings are in line with our hypothesis and with earlier work (Davis et al., 2011).

There may be several plausible interpretations of the current finding: First, incremental beliefs could “enhance” the meaning of academic setbacks. Students with low mathematics self-efficacy may have a greater likelihood of facing challenging situations, and thus they might benefit more from incremental beliefs. For example, students who lack confidence in their abilities to solve math problems would be easily threatened by math-related tasks that might result in failure (e.g., exams, classroom questioning). The more the students endorse incremental beliefs, the more they would believe that math ability is something that can be worked on, and the existing low capability does not reflect their stable traits; thus, they would value more mathematical tasks. Davis et al. (2011) found that incremental theory of mathematical ability can negatively predict helplessness only for students in the underdog position. Second, entity beliefs may guide high academic self-efficacy students to focus on developing personal competitiveness, which may evoke positive impacts. In a recent meta-analytic study, Costa and Faria (2018) found that entity beliefs were moderately associated with verbal and quantitative achievement in European samples. Thus, implicit theory may have mixed effects for students with high academic self-efficacy. Moreover, our results revealed that although high levels of academic self-efficacy weakened the positive association between the implicit theory and students’ intrinsic value, students with high academic self-efficacy reported higher scores on intrinsic value in both high and low implicit theory conditions than did students with low academic self-efficacy. These findings suggest that students who are confident in their math capability may experience more academic success and possess high levels of self-approval. Such positive experiences already serve as important resources that fuel students’ intrinsic interests in learning math (Deci et al., 1991; Ryan and Deci, 2000; Liu et al., 2018). In this case, the role of incremental beliefs becomes less important.

### Practical Implications

Previous research indicates that students with high incremental beliefs are more engaged in their learning; however, most studies focus on the role of general implicit theory (Romero et al., 2014). The present study complements the extant literature by examining how (intrinsic value) and when (low academic self-efficacy) domain-specific implicit theory is significant in promoting engagement in math among Chinese adolescent students. The results provide a better understanding of the antecedents that impact mathematics engagement based on a social-cognitive perspective (Dweck and Leggett, 1988).

Our study has important implications for educational interventions that aim at facilitating students’ mathematics engagement. First, our results highlight that incremental beliefs can provide a powerful impetus that drive students to engage in math. This is noteworthy, especially in the context of the Chinese education system, in which academic performance is the most predominant determinant for admission to Chinese universities. As the Chinese saying goes, “Score decides all your life.” Under such circumstances, obtaining good grades and maintaining high class ranking are highly emphasized. Such circumstances might work against fostering an incremental learning environment, decreasing students’ development of intrinsic value, and further reducing their willingness to engage. Therefore, school educators should be more aware of the critical role that implicit theory plays in students’ mathematics engagement, and cultivate a learning environment that emphasizes that improvements can be made through effort (Aronson et al., 2002; Blackwell et al., 2007). What calls for special attention is that implicit theory intervention may be more efficient among students in low academic performance conditions (Davis et al., 2011). To help “underdog” students escape from the vicious cycle of learned helplessness, teachers can promote their incremental beliefs by encouraging their participation and efforts, praising students for their progress, and appraising them through multiple approaches rather than focusing solely on test scores (Paunesku et al., 2015; Tarbetsky et al., 2016). In addition, another way to facilitate math engagement is to improve students’ intrinsic value. Teachers could offer more support to improve students’ understanding of math, such as interpreting basic mathematical principles using practical examples. Parents can also provide more information about their future career planning in relation to the students’ current math learning, making math relevant and rewarding for learning.

### Limitations and Future Directions

The present study has several limitations. First, our findings were based on self-reported measures, and we relied on students’ subjective report. Recent studies have suggested that males and females have different standards when self-reporting academic self-efficacy, and gender bias was observed in academic self-efficacy studies (e.g., Nielsen et al., 2018). Therefore, multimethod approaches, such as teachers’ report and parents’ report, are needed to provide more comprehensive results. Second, although a two-wave research design was adopted to reduce common method bias, causal conclusions cannot be drawn from current results (Jose, 2013). To address the question of causality, experimental, panel and multiwave longitudinal designs should be used in future research. Third, researchers have demonstrated that people can hold different theories in different domains (Burnette et al., 2013). Further studies are needed to examine the generalizability of our results in different academic domains. Moreover, it would also be interesting for future studies to bring more indicators that are closely related to math engagement into the analysis, such as prior achievement and GPA (Martin et al., 2015; Priess-Groben and Hyde, 2017). With more factors being considered, the unique relations between implicit theory and engagement would be observed.

## Conclusion

The current study investigated the underlying mechanisms accounting for the relationships between implicit theory and students’ mathematics engagement. The results showed that students’ implicit theories could positively predict engagement in math not only through a direct path but also through an indirect path via intrinsic value. Academic self-efficacy could moderate the relations between implicit theory and intrinsic value. Our study provides some guidance for educators to develop effective interventions for promoting students’ motivation and engagement in mathematics.

## Data Availability Statement

The datasets generated for this study are available on request to the corresponding author.

## Ethics Statement

The studies involving human participants were reviewed and approved by the Research Ethics Committee of the Beijing Normal University. Written informed consent to participate in this study was provided by the participants’ legal guardian/next of kin.

## Author Contributions

All authors were participants in the data collection, data analysis, and writing and revision of the manuscript and contributed to the article and approved the submitted version.

## Conflict of Interest

The authors declare that the research was conducted in the absence of any commercial or financial relationships that could be construed as a potential conflict of interest.

## Footnotes

1 http://processmacro.org
